# Alveoli, teeth, and tooth loss: Understanding the homology of internal mandibular structures in mysticete cetaceans

**DOI:** 10.1371/journal.pone.0178243

**Published:** 2017-05-19

**Authors:** Carlos Mauricio Peredo, Nicholas D. Pyenson, Mark D. Uhen, Christopher D. Marshall

**Affiliations:** 1 Department of Environmental Science and Policy, George Mason University, Fairfax, VA, United States of America; 2 Department of Paleobiology, National Museum of Natural History, Smithsonian Institution, Washington D. C., United States of America; 3 Department of Atmospheric, Oceanic, and Earth Sciences, George Mason University, Fairfax, VA, United States of America; 4 Department of Marine Biology, and Department of Wildlife and Fisheries Sciences, Texas A&M University, Galveston, TX, United States of America; University of Michigan, UNITED STATES

## Abstract

The evolution of filter feeding in baleen whales (Mysticeti) facilitated a wide range of ecological diversity and extreme gigantism. The innovation of filter feeding evolved in a shift from a mineralized upper and lower dentition in stem mysticetes to keratinous baleen plates that hang only from the roof of the mouth in extant species, which are all edentulous as adults. While all extant mysticetes are born with a mandible lacking a specialized feeding structure (i.e., baleen), the bony surface retains small foramina with elongated sulci that often merge together in what has been termed the alveolar gutter. Because mysticete embryos develop tooth buds that resorb *in utero*, these foramina have been interpreted as homologous to tooth alveoli in other mammals. Here, we test this homology by creating 3D models of the internal mandibular morphology from terrestrial artiodactyls and fossil and extant cetaceans, including stem cetaceans, odontocetes and mysticetes. We demonstrate that dorsal foramina on the mandible communicate with the mandibular canal via smaller canals, which we explain within the context of known mechanical models of bone resorption. We suggest that these dorsal foramina represent distinct branches of the inferior alveolar nerve (or artery), rather than alveoli homologous with those of other mammals. As a functional explanation, we propose that these branches provide sensation to the dorsal margin of the mandible to facilitate placement and occlusion of the baleen plates during filer feeding.

## Introduction

**Fig 1:**
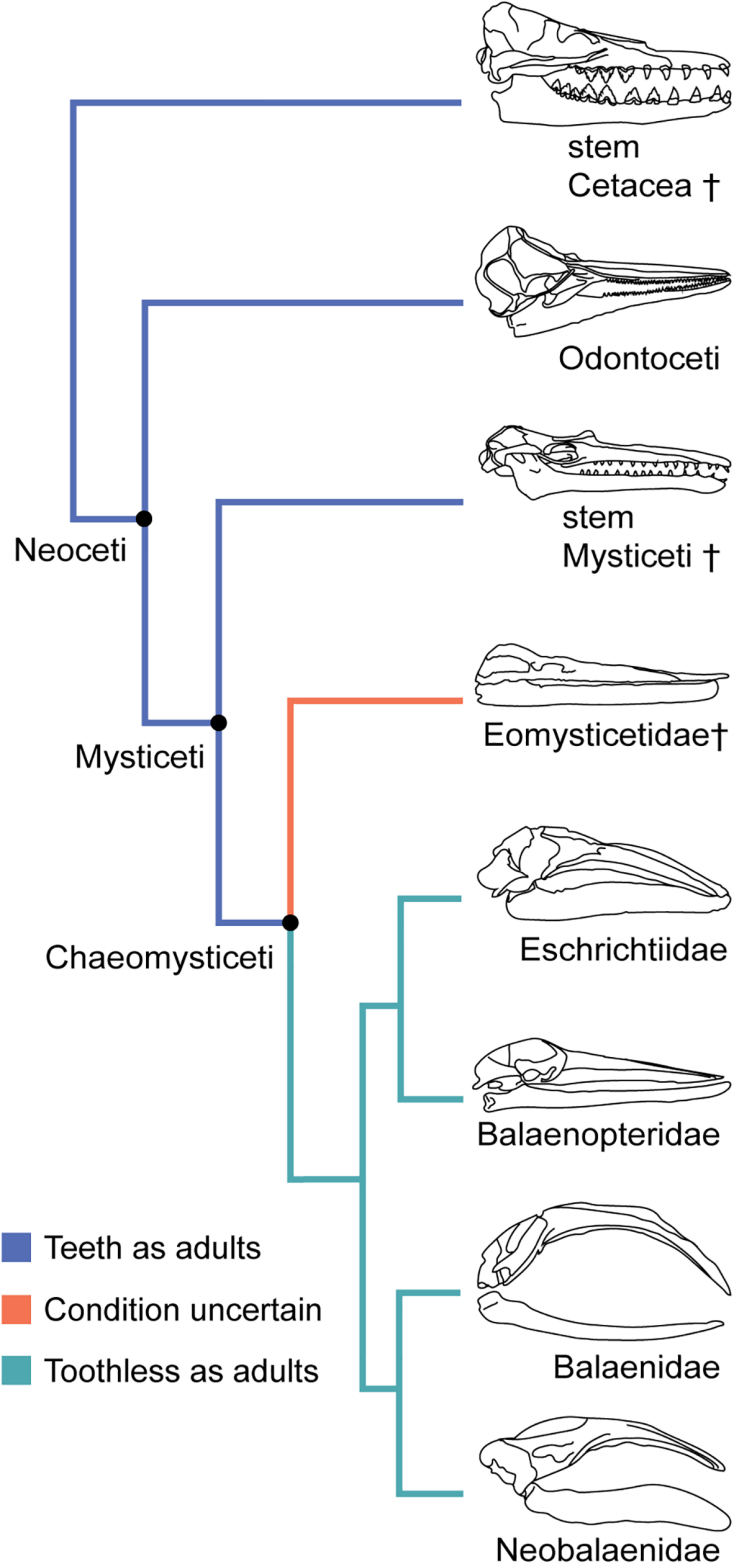
Generalized phylogeny showing the transition in mysticetes from toothed ancestors to the edentulous, baleen bearing extant taxa. Phylogeny of Cetacea with an emphasis on mysticete evolution. Blue represents the ancestral condition of adults bearing teeth. Extant mysticetes (green) are edentulous as adults, bear baleen on the palate, and dorsal foramina on the mandible. Eomysticetidae is traditionally interpreted as edentulous, though some taxa preserve possible alveoli potentially indicative of a dentition [9–11].

Mysticete cetaceans are group of marine mammals that includes the largest vertebrates in the history of the planet. Their diversity and ecological success has been linked to dramatic evolutionary shifts in their feeding mechanisms, departing from an ancestral macrophagous raptorial feeding style to filter feeding prey in bulk aggregate [1]. Mysticetes filter using elaborate racks of keratinous baleen plates, which grow from the palate to create a sieve that traps suspended prey [2–4]. Although both stem cetaceans and stem mysticetes had teeth [5], all extant mysticetes are born entirely edentulous (Fig 1). However, mysticete fetal development shows vestigial evidence of dentition, with histological studies identifying tooth buds in mysticete embryos that progress through three of the four stages of tooth development before being completely resorbed prior to birth [6–8].

**Fig 2:**
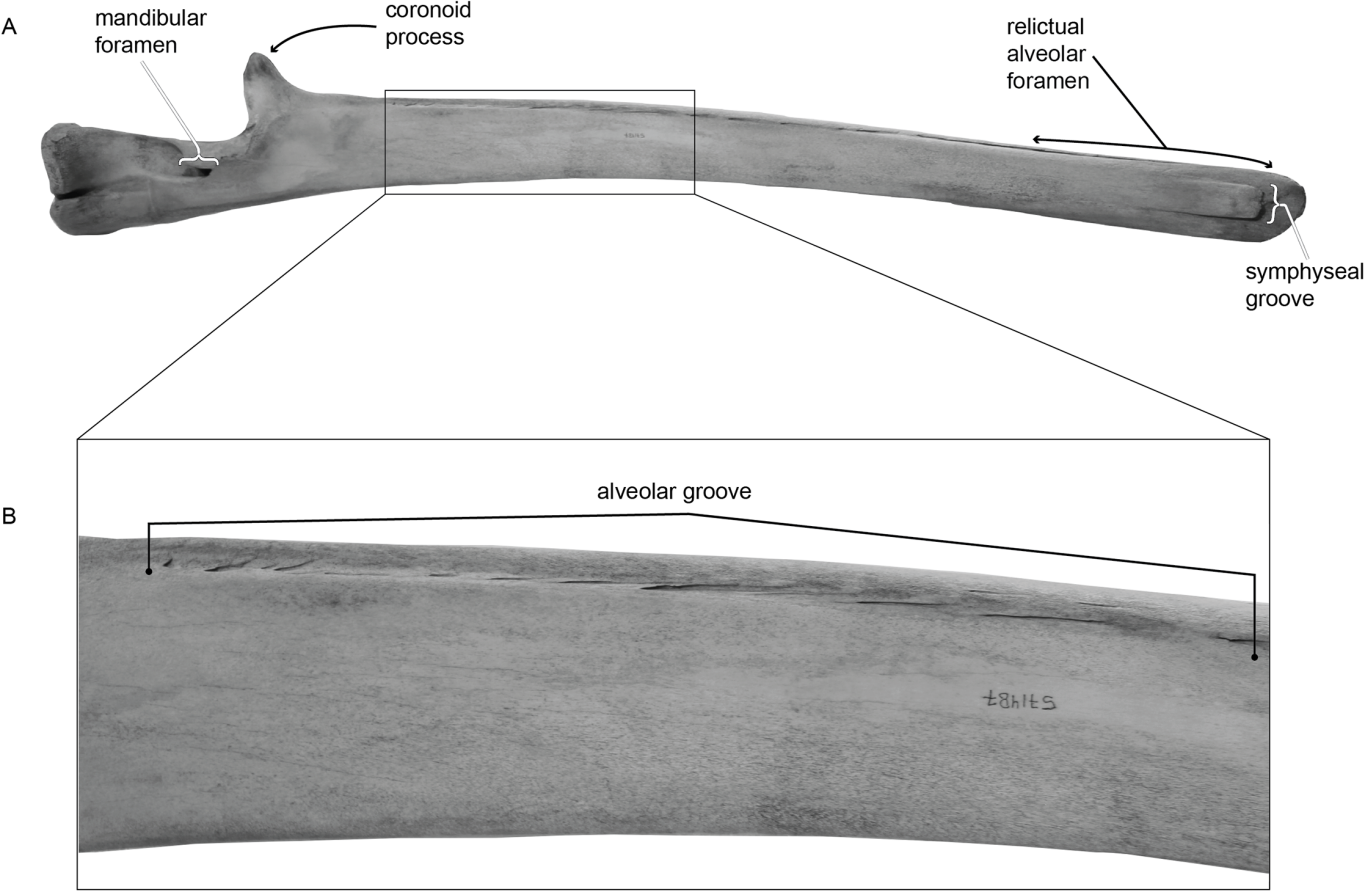
Alveolar groove on the mandible of Mysticeti. (A) Medial view of the left mandible of USNM VZ 571487, *Balaenoptera acutorostrata*. (B) Enhanced view of the selected region on panel (A), specifically highlighting the alveolar groove and associated sulci.

Because teeth are completely resorbed and baleen plates develop only on the palate, mandibles in living mysticetes lack any specialized feeding structure (i.e., neither teeth nor baleen). Nonetheless, extant mysticete mandibles preserve a series of small foramina and associated sulci (shallow grooves emanating from the foramina) on the dorsomedial surface, often appearing in close proximity to each other to form a shallow groove (Fig 2). This groove has been termed the “alveolar groove” or “alveolar gutter” by previous authors (see page 42 in [12] for a review). Personal observation indicates that the mysticete alveolar groove is present in all four families of extant cetaceans. Noticeably, the distal end of the alveolar groove is typically expanded into a deep trough, which has been termed a relictual alveolar foramen, as a proposed homolog with the first incisor alveolus [13]. Despite this association, the homology of the alveolar groove with the alveoli of other mammals has not been investigated.

Here, we test this homology by evaluating the internal morphology of mysticete mandibles in comparison with near relatives, including both terrestrial artiodactyls and other cetaceans. While an edentulous condition has evolved at least four times among mammals (Vermilingua, Pholidota, Chaeomysticeti, *Hydrodamalis*), and several times in other tetrapods (Chelonia, Neornithes), none of these independent edentulous origins are related to filter feeding, although they are associated with distinct, specialized feeding morphologies relative to near relatives [14]. Our macroscopic observations of Vermilingua, Pholidota, and *Hydrodamalis* failed to identify any such alveolar groove or foramina that may be homologous to those visible on the mandibles of Mysticeti. No mention of any such structures exists in the anatomical descriptions of anteaters [15, 16], pangolins [17], nor *Hydrodamalis* [18]. Though anteater mandibles are comparatively small to those of mysticetes, no internal evidence of such a structure is visible in the CT data reported by Endo et al. [16]. Because none of these other three edentulous taxa form a clade, neither with mysticetes nor with each other, their edentulous condition shared among them represents a homoplasy, not a homology, as defined by Hall [19, 20]. Because we aim to test the homology of dorsal foramina, we frame this study within a phylogenetic context, similar to other recent tests of homology [21–23]. A morphological comparison of the homoplasic condition of edentulousness is beyond the scope of this paper.

In terrestrial mammals, the mandibular teeth are innervated through their roots by the inferior alveolar nerve (mandibular branch of the trigeminal nerve) and are vascularized by the inferior alveolar artery (see [24, 25] for humans and [26–28] for domesticated mammals). This condition represents the ancestral condition across placental mammals. The inferior alveolar nerve enters the mandible at the posterior end through the mandibular foramen, and runs the length of the mandible ventral to the teeth. In humans, the inferior alveolar nerve supplies branches to the teeth before splitting into the mental nerve (which exits through the mental foramen to innervate the lower lip and chin) and the incisive nerve which runs as an extension of the inferior alveolar nerve and innervates the anterior teeth [24, 25]. This condition is common across placental mammals, though numerous lineages have multiple mental nerves, and therefore multiple mental foramina [26].

**Fig 3:**
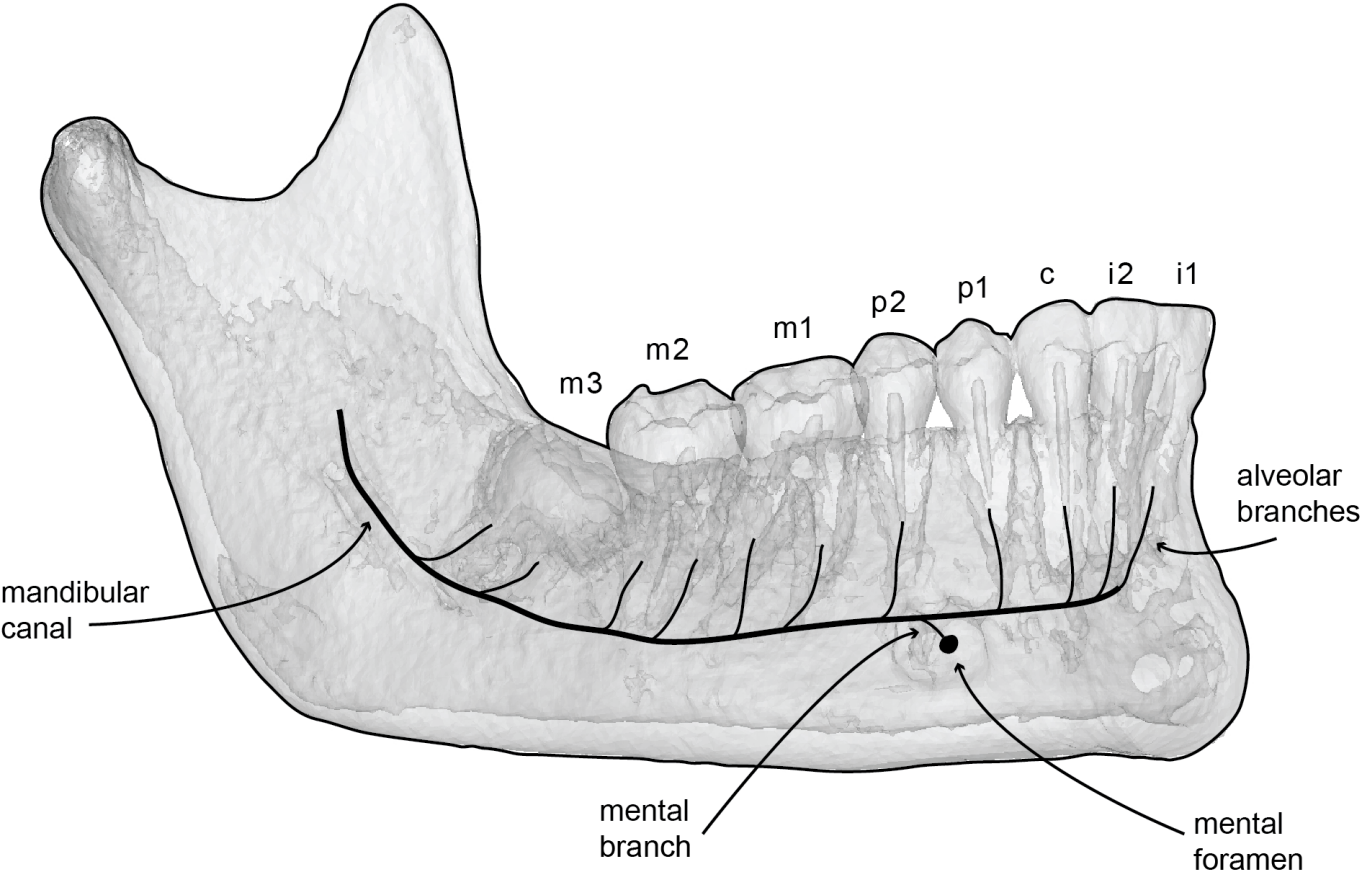
Internal canal system of the mammalian mandible. 3D model of a human mandible with line art illustration highlighting the generalized mammalian condition for innervation and vascularization of the mandible. The mandibular canal gives off dorsomedial branches to each root of each tooth, as well as mental branches that exit the mandible through mental foramina by which the lips and gums are vascularized and innervated.

In general, the mandibles of mammals possess three distinct types of canals. The mandibular canal begins its course posteriorly at the mandibular foramen and houses the inferior alveolar nerve and vasculature. Lateral branches of the inferior alveolar nerves are considered mental branches, and exit the mandible through the mental foramina. Both mental nerves and associated vasculature supply the lip and chin regions. Likewise, dorsomedial branches of the mandibular canal are considered alveolar branches, and house the nerves and vessels that supply each tooth root (Fig 3). However, several studies on humans indicate that the alveolar branches may not form traditional ossified canals in the bone but rather instead pass through trabecular bone on their way to the pulp cavity [29, 30].

The form and function of the alveolar gutter of mysticetes, and any confluent internal structures, has been largely overlooked in the literature. While some authors have commented on the size of the mandibular canal [31] or briefly described the superficial morphology of the alveolar gutters [32] in fossil taxa, no study has examined the internal morphology of mysticete mandibles and compared it to that of other mammals. Thus, the homology of the mysticete alveolar gutter to the mammalian alveolar row remains untested. Here, we test the hypothesis that the mysticete alveolar gutter and confluent internal structures is homologous to those of the mammalian alveolar row by creating 3D models of the internal anatomy of the mandibles of several members of Artiodactyla (including Cetacea). In doing so, we map the course of the mandibular canal and all its distributaries to evaluate potential homologies between the edentulous mandibles of extant mysticetes and the toothed mandibles of other mammals. Finally, we place our observations within the context of established mechanical models of bone resorption. In doing so, we call into question the homology of the mysticete alveolar gutter to the mammalian alveolar row, suggest a tentative alternative, and outline further directions by which they may be tested.

## Materials and methods

### Institutional abbreviations

Institutional Abbreviations. DFO, collections in the Department of Zoology, University of British Columbia, Vancouver, Canada; SDSNH, San Diego Natural History Museum, San Diego, California, U.S.A.; USNM, Departments of Paleobiology and Vertebrate Zoology (Division of Mammals), National Museum of Natural History, Smithsonian Institution, Washington, District of Columbia, U.S.A.

### Taxonomic sampling

To build a sample dataset with a phylogenetic context, we sampled seven taxa within Artiodactyla that we scanned using computed tomography (CT) to create 3D models of the external bone surface and internal morphology. Our sample included wild boars (*Sus scrofa*), white-tailed deer (*Odocoileus virginianus*), a stem cetacean (*Zygorhiza kochii*), bottlenose dolphins (*Tursiops truncatus*) and three mysticetes, minke (*Balaenoptera acutorostrata*), humpback (*Megaptera novaeangliae*), and pygmy right whale (*Caperea marginata*) (see Table 1). Scans of DFO 2408 were provided courtesy of Jeremy A. Goldbogen and scans of SDSNH 21212 were obtained via Digimorph courtesy of Timothy Rowe.

**Table 1:** List of specimens scanned, location of scan, and number of CT slices.

Taxon	Specimen	Scanning Facility	Number of Slices
*Sus scrofa*	USNM VZ 260907	Smithsonian Institution Bio-Imaging Research (SIBIR) Center in the Department of Anthropology at USNM	2,498
*Odocoileus virginianus*	USNM VZ 118627		2,014
*Zygorhiza kochii*	USNM PAL 11962		5821
*Balaenoptera acutorostrata*	USNM VZ 571847		7564
*Caperea marginata*	USNM VZ 550146		7915
*Tursiops truncatus*	SDSNH 21212	High-Resolution X-ray Computed Tomography Facility at the University of Texas at Austin	628
*Megaptera novaeangliae*	DFO 2408	Vancouver General Hospital	2010

Our taxonomic selections represent the diversity of feeding morphologies within Cetacea and terrestrial artiodactyls. Despite the upper size limitation for mysticete mandibles [33], we include three different mysticete species to ensure that our findings were not merely limited to a single taxon. The inclusion of *T*. *truncatus* and *Z*. *kochii* represent the apomorphic homodont condition of odontocetes and the plesiomorphic heterodont dentition of stem cetaceans, respectively. Lastly, we included terrestrial artiodactyls both with and without a fused mandibular symphysis.

We scanned our sample in the Smithsonian Institution Bio-Imaging Research Center in the Department of Anthropology at the National Museum of Natural History with a Siemens Somatom Emotion 6 at slice thickness of 0.63 mm (which results in a three-dimensional reconstruction increment of 0.30 mm). The resultant DICOM files were processed by loading image files in Mimics Innovation Suite 19 (Materialise NV, Leuven, Belgium), and a mask was created based on the threshold of bone, relative to the nominal density of air. We then created a three-dimensional (3D) object from this mask, and exported the resultant file as a binary STL, which was opened in Materialise for final imaging edits and segmentation to create 3D models of the external and internal morphology of the mandibles. We made separate models for the surface of the bone, the mandibular canal, the mental branches, and the alveoli. For specimens with teeth, we modeled dentition within the alveoli to eliminate the subjectivity of separating tooth roots from tight articulation with the alveoli in the mandibles.

We collected all measurements digitally from the 3D files and followed terminology used by Mead and Fordyce [12]. For all specimens, we modeled a single mandible; for specimens preserving both mandibles in articulation, we restricted the model to only a single side and terminated at the symphysis.

## Results/Morphological descriptions

### Sus scrofa

**Fig 4:**
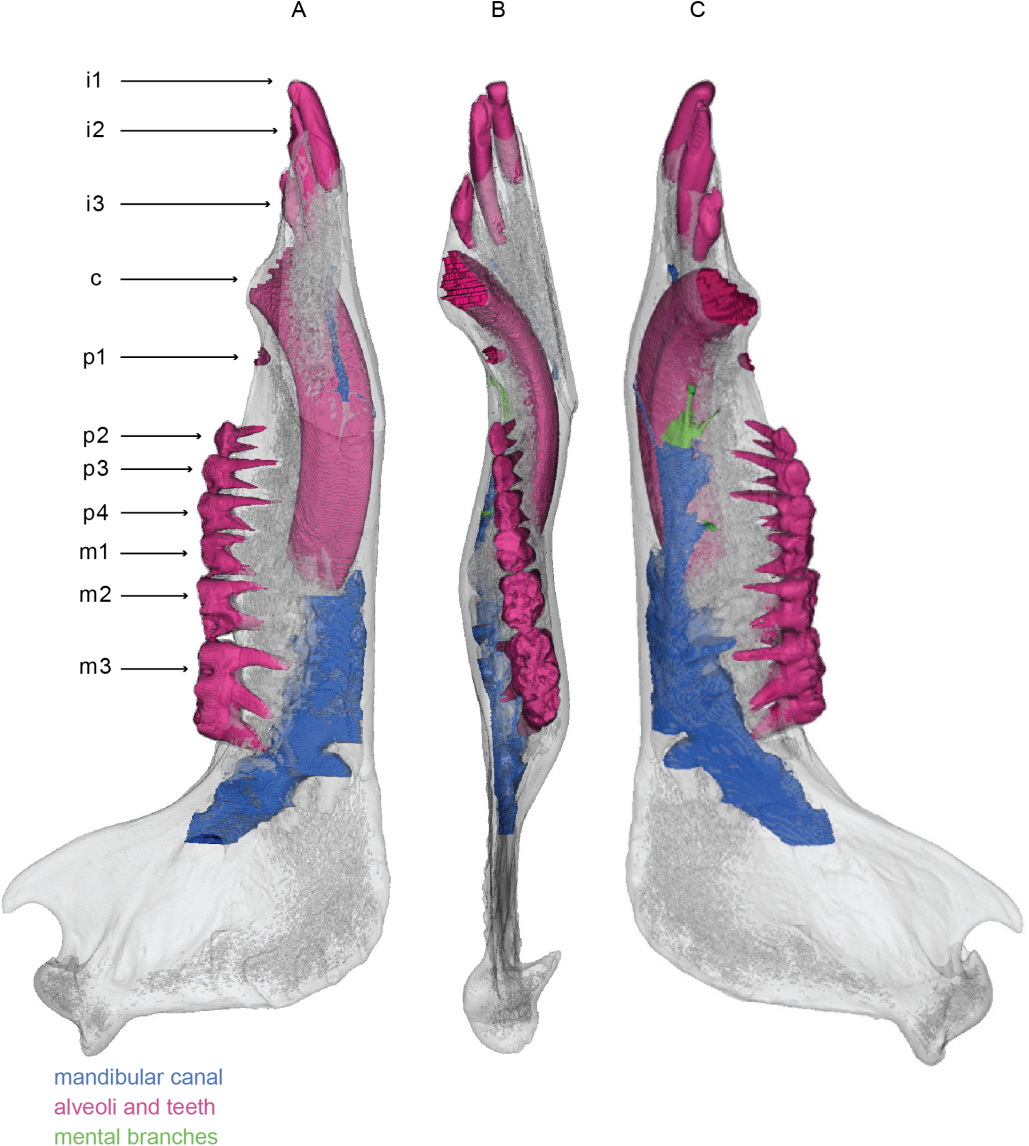
Mandible of *Sus scrofa*. 3D model of the left mandible of *Sus scrofa* (USNM VZ 260907). 3D model figured in medial (A), dorsal (B), and lateral (C) views. White represents the external bony surface, pink the alveoli and teeth, green the mental branches, and blue the mandibular canal.

The mandible of *Sus scrofa* is anteroposteriorly short and transversely thin compared to those of extant Cetacea (Fig 4). In dorsal view, the mandible is thin posteriorly, with a blade-like coronoid process and a thickened, knob-shaped mandibular condyle. The mandible widens anteriorly to accommodate the teeth but remains relatively narrow overall. In lateral view, the dorsal and ventral margins are parallel from the angle with the ramus to the level of the canine, where the ventral margin of the body sharply angles dorsally to meet the dorsal margin. Posteriorly, the body and the ramus create a gently curved angle at approximately 135 degrees. Internally, the mandibular canal lies near the ventral margin of the mandibular body.

Wild boars possess three incisors, an enlarged canine, four premolars, and three molars. The roots of the molars reach deep into the body of the mandible, so that they nearly penetrate the mandibular canal. Externally, the canine curves medially from its apex to the mandibular body, and continues internally within the body of the mandible. The roots of the premolars, especially the first, are drastically shortened, unlike those of any other mammal studied here, suggesting that they are shortened to accommodate the root of the canine. This deep penetration by the root of the canine also results in a drastic diminishing of the mandibular canal, which becomes nearly impossible to delineate as it passes around the root. The incisors possess shallow roots, and are strongly oriented anteriorly. The tilt of the incisors mirrors the anterior margin of the body of the mandible. Well-defined pulp cavities of all 11 teeth are clearly visible in the CT data.

The mandibular canal begins at the mandibular foramen, which is located approximately mid-way along the ramus. It descends at a 45-degree angle within the ramus and then expands dorsoventrally as it transitions into the horizontal body of the mandible. As it approaches the level of the canine, the mandibular canal becomes diminished and compressed laterally and ventrally. Here, the mandibular canal splits into a lateral branch that continues to the anterior-most mental foramen, and a ventral continuation of the main canal. The ventral branch becomes extremely compressed, both dorsoventrally and transversely, so that it is barely patent. This miniscule continuation of the mandibular canal turns ventrally and medially, so that it passes below the massive root of the canine. Anterior to the canine, the mandibular canal remains extremely compressed and terminates at the distal end of the mandible where it communicates with the first incisor.

Three mental foramina can be observed on the external surface of the mandible of *Sus scrofa*. However, only two of these were large enough in diameter to be visible in the CT data. Of these, the posterior-most is small, directed laterally, and short in overall distance. The anterior-most is robust, especially at its origin where it branches from the mandibular canal. It is more anteriorly directed and bifurcates, resulting in two separate external openings. No individual alveolar branches to the tooth roots are identifiable. However, it is possible that the branches are too small to be observed at this scan resolution. Another possibility is that the alveolar branches of the inferior alveolar nerve and artery are passing through the trabecular bone without forming ossified or bony channels as often observed in humans [30, 34].

### Odocoileus virginianus

**Fig 5:**
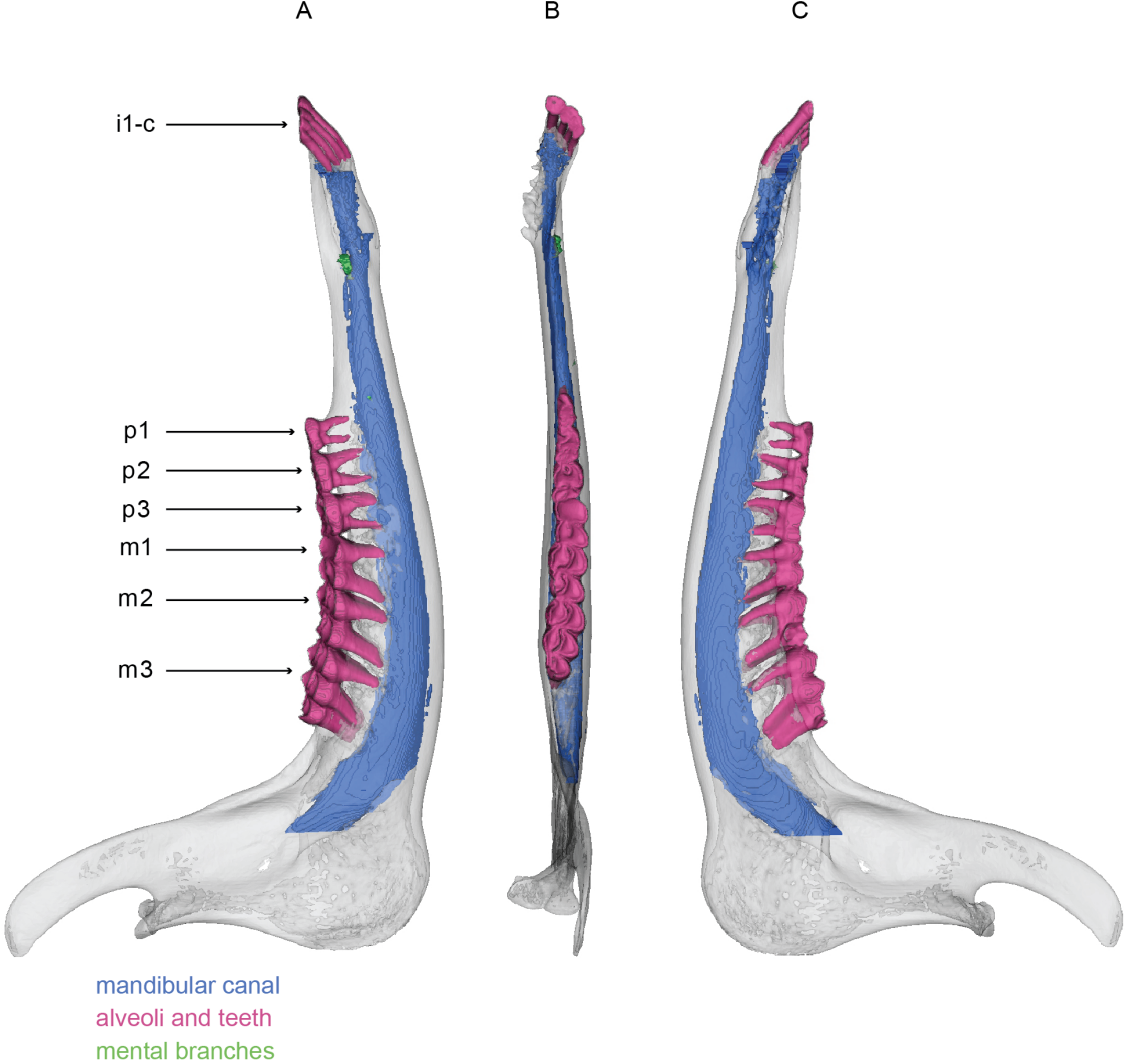
Mandible of *Odocoileus virginianus*. 3D model of the right mandible of *Odocoileus virginianus* (USNM VZ 118627). 3D model figured in lateral (A), dorsal (B), and medial (C) views. White represents the external bony surface, pink the alveoli and teeth, green the mental branches, and blue the mandibular canal.

The mandible of *Odocoileus virginianus* is similar to that of *Sus scrofa* in being anteroposteriorly short compared to mysticete cetaceans (Fig 5). In dorsal view, it is transversely thin, blade-like and just wide enough transversely to accommodate the cheek teeth. In lateral and medial views, the ramus angle is 90 degrees to the mandibular body. The coronoid process is elevated and exhibits a lobate apex that bears a slight posterior inclination. The mandibular condyle rises to about two-thirds the height of the coronoid process, well above the occlusal plane, and ends as a thickened, transversely widened knob. The body of the mandible exhibits nearly parallel dorsal and ventral margins (excluding the dentition) for most of its length, though distally the ventral margin takes on a gentle dorsal angle to rise and meet the dorsal margin. Unlike *Sus scrofa*, the mandibular symphysis of *Odocoileus virginianus* is not fused, though there is articulation. Each distal hemi-mandible is pitted and rugose, indicative of where soft tissue attached to supplement the loose articulation of each hemi-mandible.

The mandible of *Odocoileus virginianus* has three incisors, one canine, three premolars, and three molars. As in *S*. *scrofa*, the incisors are closely spaced together. However, unlike *S*. *scrofa*, the canine lies in sequence with the incisors and bears no signs of enlargement. A large diastema is present between the canine to the first premolar. All the teeth, and most especially the cheek teeth, are deeply rooted and penetrate nearly to the mandibular canal. As in *S*. *scrofa*, the incisors show a marked anterior inclination that mirrors the ventral margin of the body of the mandible.

The mandibular canal of *Odocoileus virginianus* begins at the mandibular foramen about a third of the way up the ramus. At its origin, it is nearly dorsally oriented but quickly angles ventrally and descends within the mandible at a 45 degree angle. It quickly and sharply turns to adopt the anteroposterior orientation confluent with the body of the mandible. The mandibular canal retains a relatively consistent dorsoventral height for most of its length, becoming restricted distally where it approaches the mandibular symphysis at the anterior termination of the mandible. Here, the mandibular canal narrows and passes under the canine and incisors before terminating at the first incisor. The drastic reduction in size and sharp change of direction observed in *Sus scrofa* are not present in *Odocoileus virginianus*, supporting the interpretation that this morphology is a result of the enlarged canine and its deeply penetrating root.

The mandible of this specimen of *Odocoileus virginianus* has two mental foramina. As with *S*. *scrofa*, the first of the two mental foramina is transversely oriented and very small, both in length and in diameter. The second of the two shows a more anterior inclination and is slightly larger in diameter, but does not approach the relative size, nor does it exhibit the bifurcation seen in *S*. *scrofa*. As with the mandible of *S*. *scrofa*, no individual alveolar branches of the mandibular canal are visible. Again, this may be attributable to scan resolution or may indicate that the alveolar branches are passing through trabecular bone rather than forming ossified canals.

### Zygorhiza kochii

**Fig 6:**
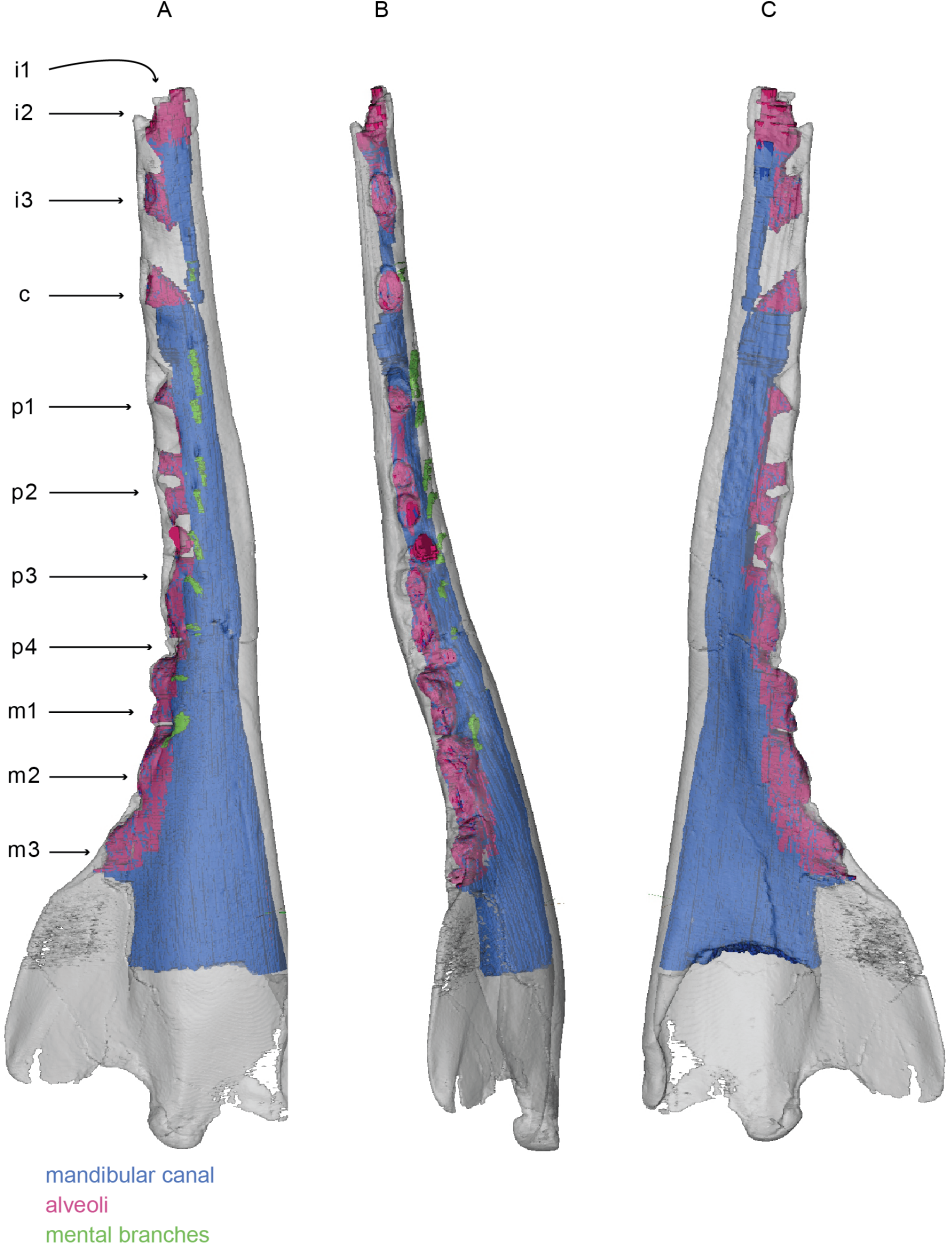
Mandible of *Zygorhiza kochii*. 3D model of the right mandible of *Zygorhiza kochii* (USNM PAL 11962). 3D model figured in lateral (A), dorsal (B), and medial (C) views. White represents the external bony surface, pink the alveoli, green the mental branches, and blue the mandibular canal.

The mandible of the stem cetacean *Zygorhiza kochii* (USNM PAL 11962) is anteroposteriorly elongated compared to *Sus scrofa*, but still relatively short compared to extant Mysticeti (Fig 6). The mandibular canal is narrow transversely and, unlike the relatively straight mandible of *Sus scrofa*, preserves a medial bowing characteristic of basilosaurids [35]. The coronoid process is thin and blade-like in dorsal view, is long anteroposteriorly, and is markedly elevated above the mandibular condyle. The mandibular condyle is a short knob oriented posteriorly, not dorsally as in *Sus scrofa*. A deep, semicircular mandibular notch can be observed between the coronoid process and the mandibular condyle. A thorough description of the external morphology of the mandibles of basilosaurids can be found in Uhen [35].

The teeth of USNM PAL 11962 were not scanned with the mandible, and are not described here. However, *Zygorhiza kochii* possesses three incisors, one canine, four premolars, and three molars. All the alveoli associated with this dentition are preserved, except for the alveolus of the first incisor. The distal tip of the mandible is broken in this specimen, leaving only a small amount of the alveolus visible. Additionally, only a single alveolus is preserved at the level of the first premolar, despite the fact that the adult lower first premolar of *Zygorhiza kochii* is double rooted. Examination of the lower first premolar shows that, while double rooted, the roots are tightly appressed yet occupy a single alveolus lacking a bony septum between the roots. All the alveoli are deep, suggesting that the roots of the teeth should have penetrated the mandibular canal. Bony separations between the mandibular canal and the alveoli are not visible. *Zygorhiza kochii* possesses single rooted incisors and canines, and double rooted cheek teeth. The anterior alveoli are moderately spaced, but the posterior alveoli, especially those of the molars, are tightly appressed against each other. While individual alveoli are more visible on the surface of the mandible, internally the proximal alveoli are so closely appressed that there is no bony division between them. The result is that the internal space between the molars appears as a single, continuous alveolar cavity that would have housed six tooth roots (three teeth).

The mandibular foramen is greatly expanded dorsoventrally, which is typical for stem cetaceans, and is associated with improved underwater hearing [36–38]. The result is that the diameter of the mandibular canal is large and dorsoventrally expanded at the mandibular foramen. Moving anteriorly, the ventral surface of the canal remains horizontal, but the dorsal surface of the canal parallels the dorsal margin of the body of the mandible, resulting in the canal becoming dorsoventrally restricted and becomes overall cylindrical in shape and it courses anteriorly. At the level of the canine, the mandibular canal becomes even more reduced and is dorsoventrally narrow where it passes ventral to the incisors. The break at the anterior tip of the mandible obscures the termination of the mandibular canal, though what is preserved indicates that it likely would have continued to a point below the first incisor as observed in *Sus scrofa* and *Odocoileus virginianus*.

In total, *Zygorhiza kochii* has ten mental branches. The lateral extension of all the mental foramina is relatively short, since the mandibular canal occupies much of the transverse width of the mandible. Each mental foramen is already situated near the lateral margin. The posteriormost mental foramen is positioned dorsolaterally on the mandible near the dorsal margin. Moving anteriorly, subsequent mental foramina gradually appear lower on the lateral mandibular surface, so that the anteriormost foramen lies near the ventral margin in lateral view. The mental foramina also gradually develop an anterior tilt, with the posteriormost foramen being laterally directed and the anteriormost foramen angling approximately 15 degrees from the sagittal plane. No individual alveolar branches are preserved, as the alveoli are deep and wide so that they penetrate into mandibular canal. Thus, the alveolar branches feeding the pulp cavity of the roots would not be preserved as ossified channels.

### Tursiops truncatus

**Fig 7:**
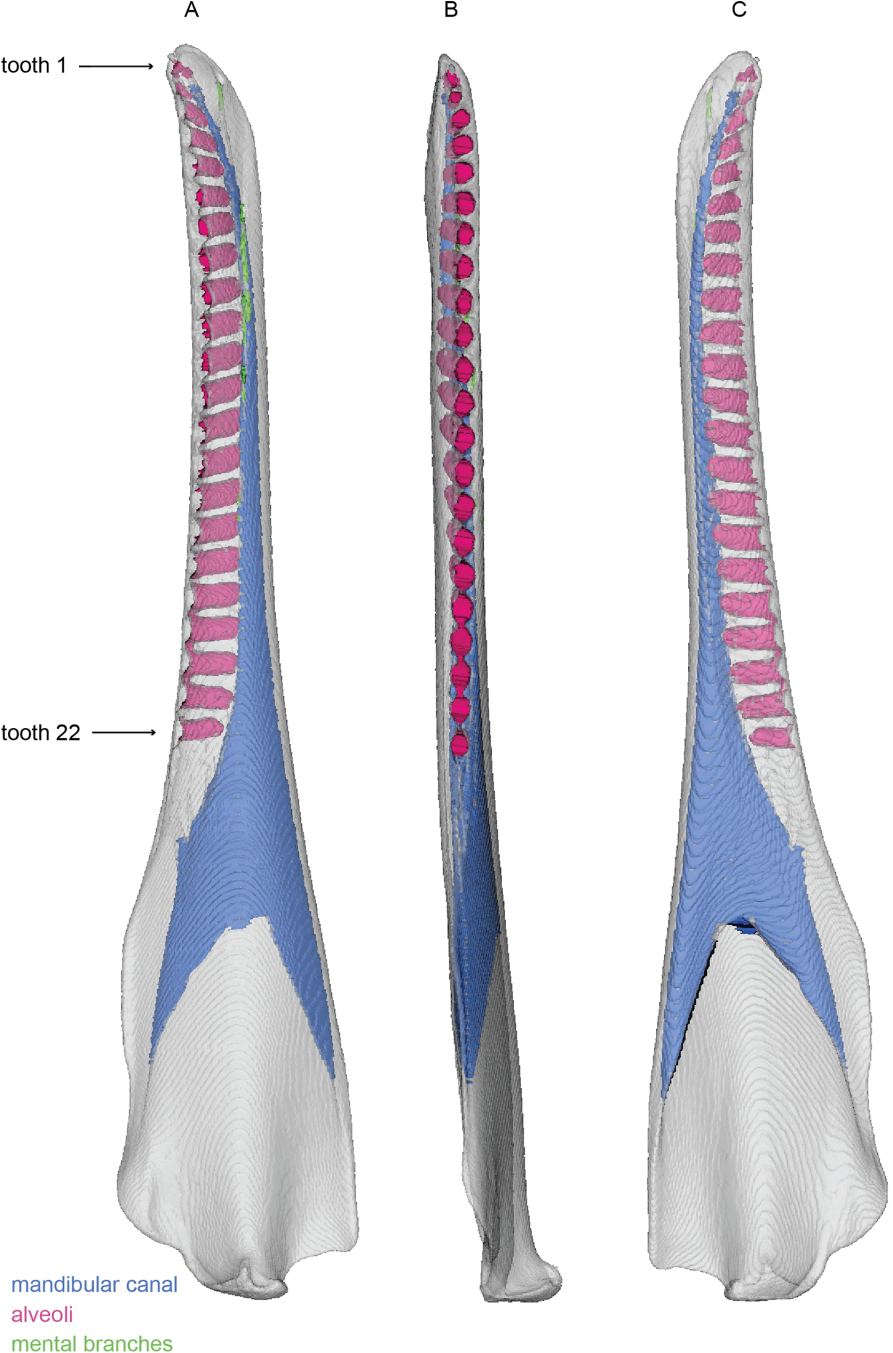
Mandible of *Tursiops truncatus*. 3D model of the right mandible of *Tursiops truncatus* (SDSNH 21212). 3D model figured in lateral (A), dorsal (B), and medial (C) views. White represents the external bony surface, pink the alveoli, green the mental branches, and blue the mandibular canal.

The mandible of *Tursiops truncatus* is anteroposteriorly elongate compared to that of *Sus scrofa* (Fig 7). It is almost entirely straight in dorsal view, possessing neither the medial bowing observed in *Zygorhiza kochii* nor the lateral bowing of mysticetes. The coronoid process is short with a blunt, rounded tip. The mandibular condyle is directed posteriorly, in line with the mandibular body. There is almost no mandibular notch; instead the coronoid process transitions to the mandibular condyle at an angle of approximately 100 degrees. In lateral view, the mandible is dorsoventrally tallest posteriorly between the coronoid process and the mandibular foramen. Anterior to this position, the mandible abruptly narrows dorsoventrally and then levels off to establish parallel dorsal and ventral margins. At the anterior tip, the ventral margin rises sharply at a 45 degree angle to join the dorsal margin, resulting in a pointed symphysis similar to *Sus scrofa*.

The alveoli of *Tursiops truncatus* are present as evenly spaced cylinders. This even spacing is consistent with the single rooted teeth of odontocetes. A bony septum separates each individual alveolus, unlike *Zygorhiza kochii* that showed no septa between the posterior teeth. The alveoli extend just deep enough to contact the mandibular canal, in contrast to the deep penetration of the canal observed in *Zygorhiza kochii*. Notably, the five anterior-most teeth, and the three posterior-most teeth are not as deeply rooted as the middle fourteen teeth. In total, this specimen of *Tursiops* has 22 alveoli per quadrant, though this condition can vary among individuals.

The mandibular foramen in *Tursiops truncatus* is dorsoventrally expanded and houses an intra-mandibular fat body [36], bounded laterally by the pan bone, and communicates directly with the tympanoperiotic complex of the middle ear. This fat body has a similar density of seawater and impedance matching that is critical to the reception of sound for echolocation [39, 40]. The diameter of the mandibular canal is very large at the mandibular foramen. The mandibular canal narrows anteriorly and is positioned ventrally within the mandible. The canal is still expanded where it passes ventral to the three posterior-most teeth. However, by the position of the fourth tooth, it has narrowed considerably and remains narrow as it passes ventral to the remainder of the tooth row. Distally, it angles sharply and rises dorsally within the mandible but remains ventral the alveoli. Therefore, the mandibular canal always remains close to ventral margin of the alveoli, even at the anterior and posterior regions where the alveoli are less deeply rooted. Posteriorly, this is achieved because the mandible is still expanded dorsoventrally from its origination point at the mandibular canal. Anteriorly, this close approximation to the alveoli is achieved by a dorsal deflection of the mandibular canal (rather than a re-expansion of the canal).

In total, *Tursiops truncatus* has four mental foramina. All four are narrow in diameter. The posterior-most mental foramen is oriented laterally, and is therefore short. The subsequent three foramina are oriented increasingly anteriorly, so that the anteriormost lies at an angle approximately 5 degrees from the sagittal plane. This strong anterior orientation results in progressively greater foramina length that are much longer than those of *Sus scrofa* and *Zygorhiza kochii*. No alveolar branches are visible. In this aspect, the mandible of *Tursiops truncatus* closely resembles that of *Zygorhiza kochii*, with the alveoli being deep enough to penetrate the mandibular canal. Thus, there are no bony divisions between the alveoli and the mandibular canal and the alveolar branches are likely not preserved as ossified canals.

### Mysticete mandibles

**Fig 8:**
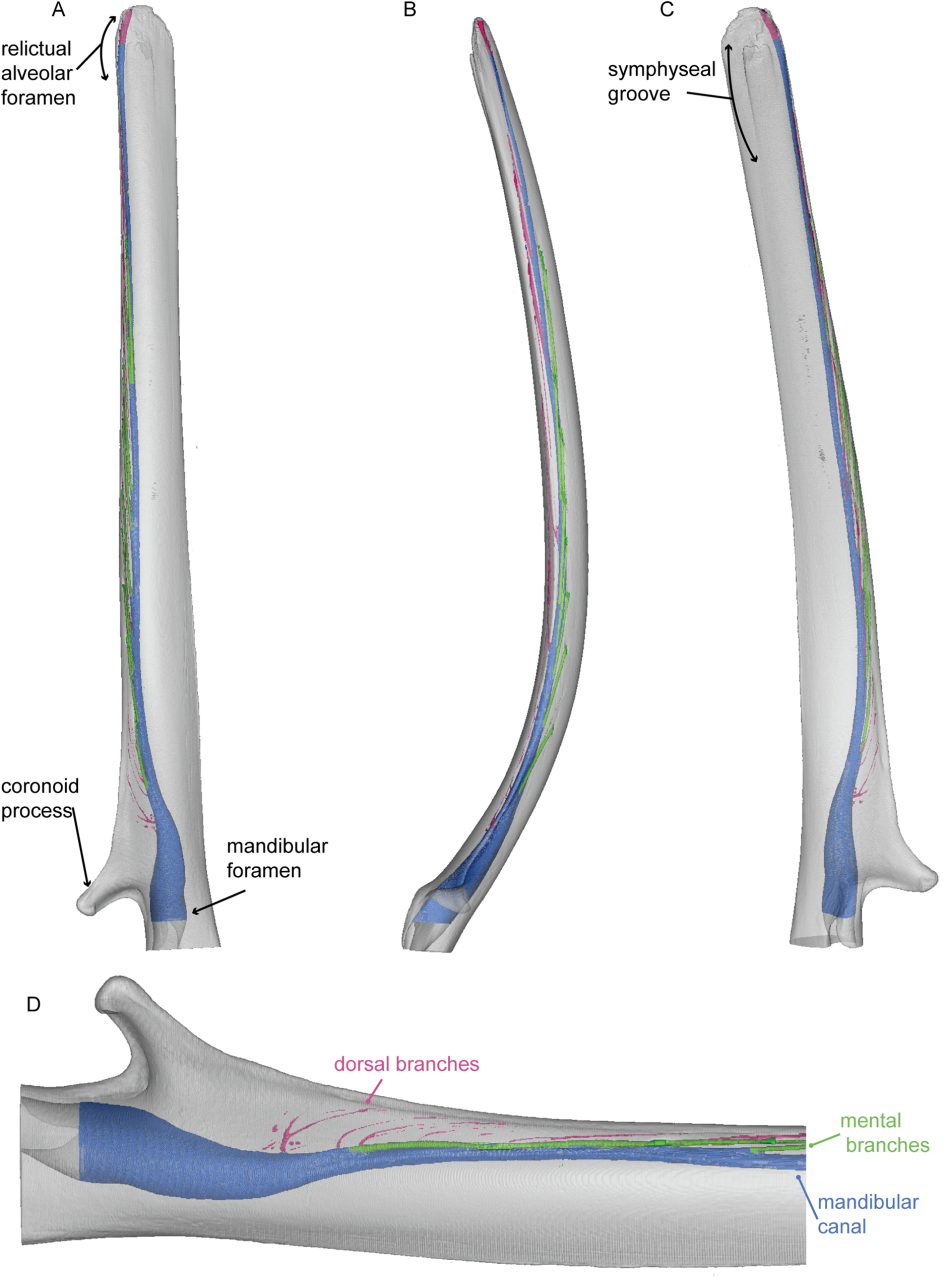
Mandible of *Balaenoptera acutorostrata*. 3D model of the right mandible of Balaenoptera acutorostrata (USNM VZ 571847). 3D model figured in lateral (A), dorsal (B), and medial (C) views. (D) represents a magnification of the posterior end of the mandible in lateral view, highlight dorsal branches from the mandibular canal as distinct from mental branches. White represents the external bony surface, pink the dorsal branches, green the mental branches, and blue the mandibular canal.

**Fig 9:**
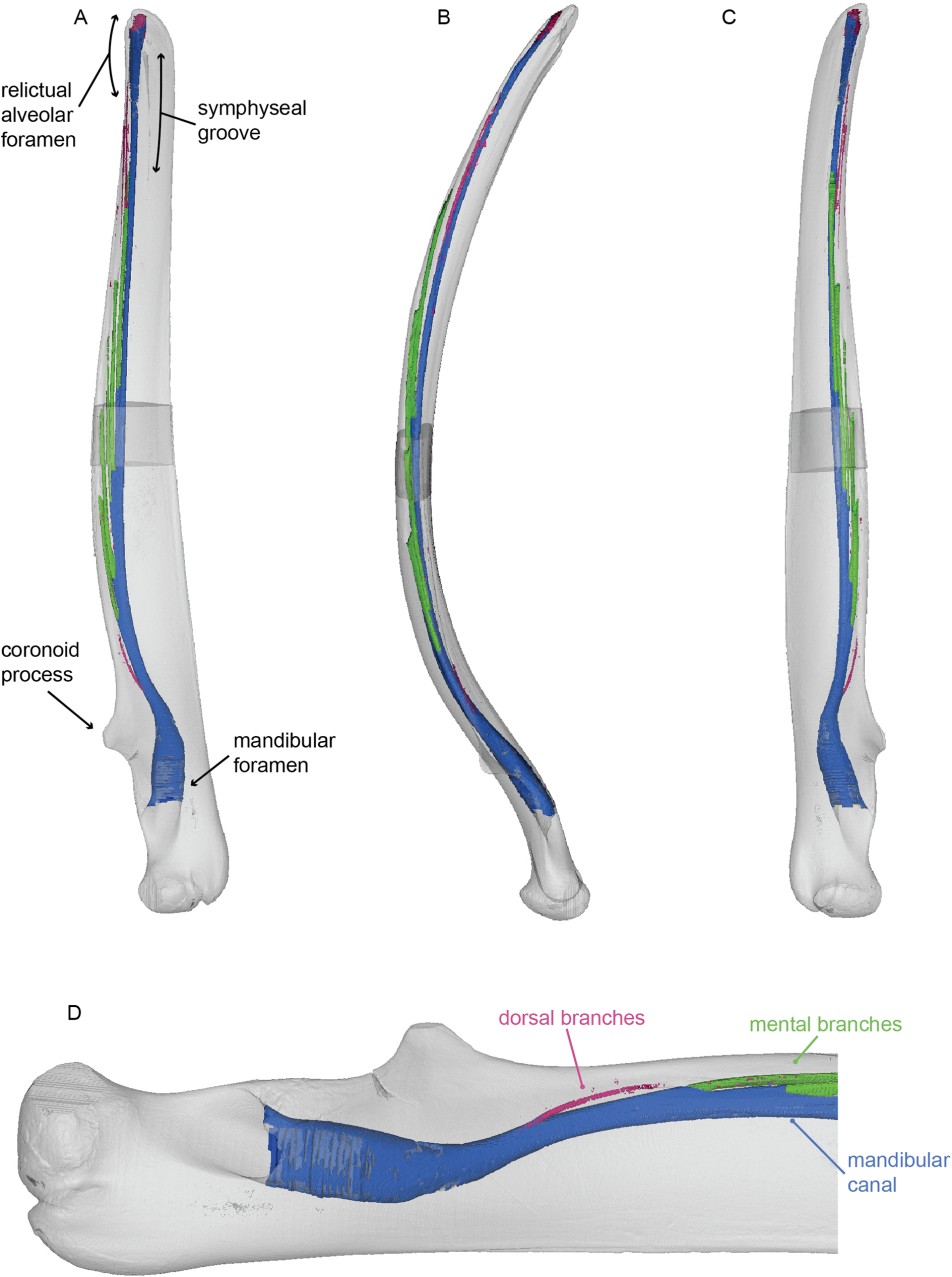
Mandible of *Megaptera novaeangliae*. 3D model of the left mandible of *Megaptera novaeangliae* (DFO 2408). 3D model figured in medial (A), dorsal (B), and lateral (C) views. (D) represents a magnification of the posterior end of the mandible in medial view, highlight dorsal branches from the mandibular canal as distinct from mental branches. White represents the external bony surface, pink the dorsal branches, green the mental branches, and blue the mandibular canal.

**Fig 10:**
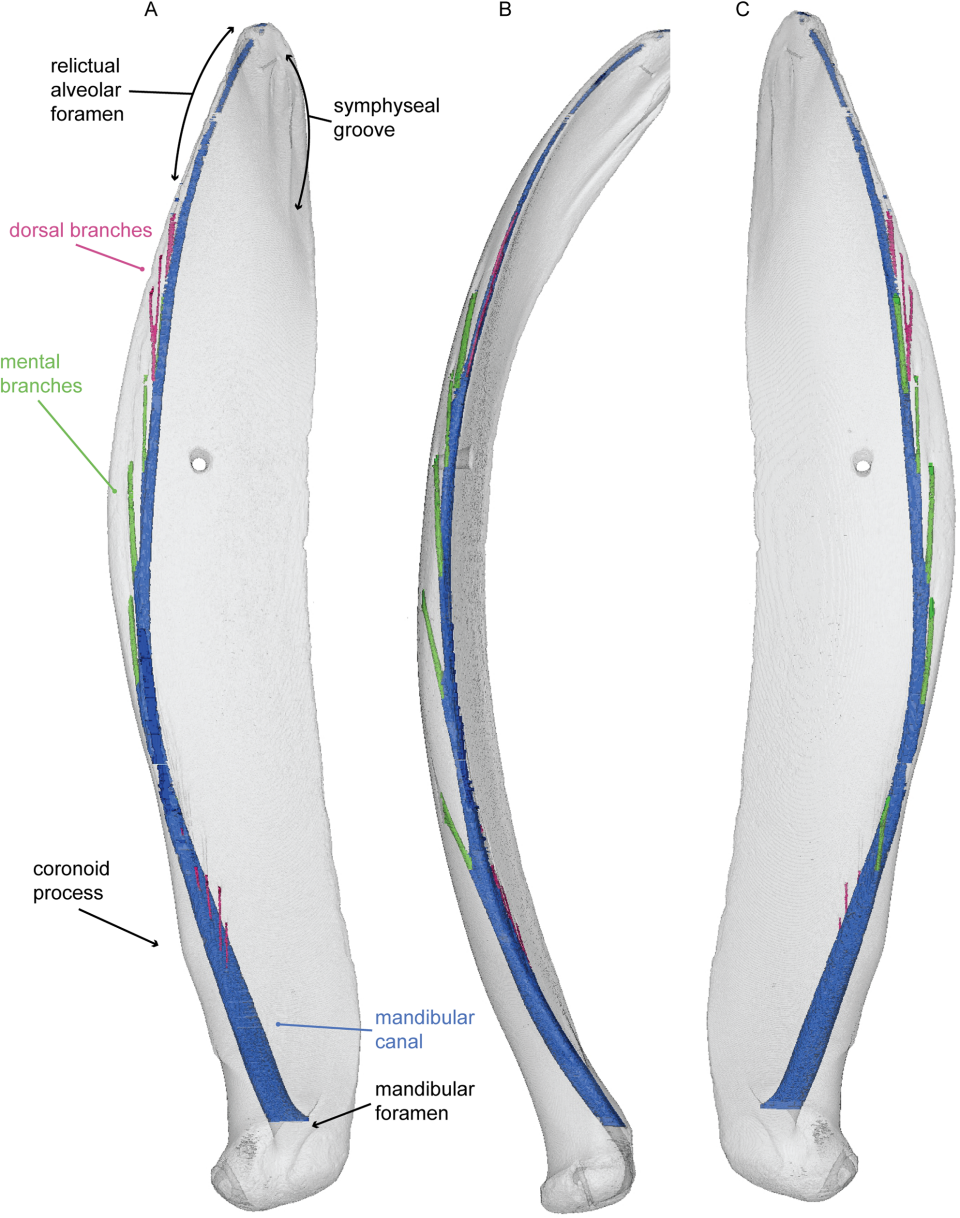
Mandible of *Caperea marginata*. 3D model of the left mandible of *Caperea marginata* (DFO 2408). 3D model figured in medial (A), dorsal (B), and lateral (C) views. White represents the external bony surface, pink the dorsal branches, green the mental branches, and blue the mandibular canal.

All three mysticete mandibles are anteroposteriorly elongate and laterally bowed (Figs 8–10). Both conditions represent innovations associated with increased oral volume for bulk feeding [3]. Each mandible has expanded articular condyles directed posteriorly, in line with the mandibular body. *Balaenoptera acutorostrata* has a relatively high, rounded coronoid process, whereas *Megaptera novaeangliae* has a relatively low, small coronoid process that extends from the dorsal margin of the body of the mandible as a small knob. *Caperea marginata* has almost no coronoid process at all; it preserves as merely a slight swelling of the dorsal margin of the mandible. In lateral view, the body of the mandible of *Balaenoptera acutorostrata* has parallel dorsal and ventral margins. The body of the mandible of *Megaptera novaeangliae*, in contrast, bears a straight ventral margin but a slightly sinusoidal dorsal margin, so that the mandible is expanded dorsoventrally mid-way along its length and constricted at the distal end. The body of the mandible of *Caperea marginata* resembles that if *Megaptera novaeangliae*, though is even more extreme still in dorsal curvature. The mandible of *Caperea marginata* differs notably from the other two in being extremely compressed laterally so that it is much thinner in cross section as opposed to the rounded or tear dropped cross section of rorquals. It further differs from the rorqual mandibles by lateral rotation of the distal tip common of balaenids and neobalaenids. All three mandibles possess a symphyseal groove on the medial surface, which is typical of crown and some stem Mysticeti [1]. Each mandible has a relatively small mandibular foramina comparable in diameter to that of *Sus scrofa* rather than to the enlarged mandibular foramina of *Zygorhiza kochii* or *Tursiops truncatus*. The mandibular foramina of *Balaenoptera acutorostrata* and *Caperea marginata* are oriented directly posteriorly, whereas the mandibular foramen of *Megaptera novaeangliae* is deflected dorsally approximately 20 degrees.

All three mandibular canals of are cylindrical and are proportionately narrower dorsoventrally than those of *Zygorhiza kochii* and *Tursiops truncatus*. Those of the two rorquals narrow drastically as they pass beneath the coronoid process and transition into the body of the mandible. Just anterior to the coronoid process, the mandibular canals deflect dorsally, so that the canals lie dorsally within the mandible, in close approximation to the dorsal surface. This condition differs starkly from all other mandibles observed, where the mandibular canal lies ventrally, close to the ventral surface in the mandibular body. In each mandible, the canal continues to rise distally so that near the terminus they breach the dorsal external surface to create a deep, open gutter that runs to the distal tip of each hemi-mandible. This gutter has been termed the relictual alveolar foramen by Pyenson et al. [13], who suggested that it may be homologous with the alveolus for the first incisor in terrestrial mammals.

**Fig 11:**
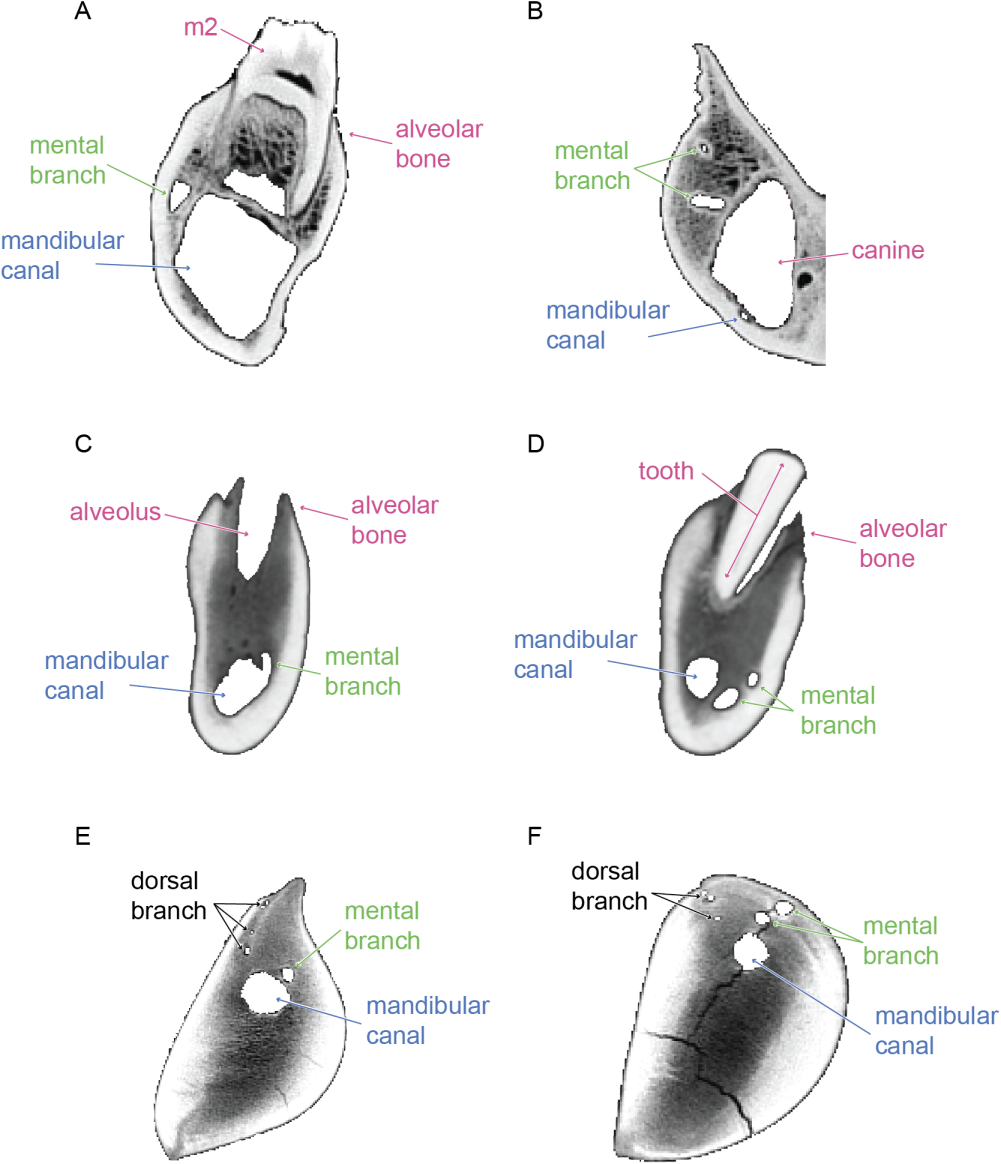
Internal morphology of mysticete mandibles are unique compared to those of toothed mammals. Cross sections of the left mandible of *Sus scrofa* (A, B), the right mandible of *Tursiops truncatus* (C, D), and the right mandible of *Balaenoptera acutorostrata* (E, F). Label colors follow the scheme of previous figures: pink represents alveoli and/or teeth, green the mental branches, and blue the mandibular canal. Here, the dorsal branches observed in *Balaenoptera acutorostrata* are labeled in black to reflect the uncertainty of their homology to alveolar branches of the toothed taxa observed.

The mandibles of *Balaenoptera acutorostrata*, *Megaptera novaeangliae*, and *Caperea marginata* have 11, 5, and 5 mental branches respectively. In all three, the mental branches are relatively wide in their diameter, especially posteriorly where they can sometimes rival the size of the mandibular canal (Fig 11). In all three taxa, the mental branches are angled sharply anteriorly. Therefore, the branches are both absolutely and proportionally the longest of the taxa in this study. Consistent with the position of the mandibular canal, the mental branches of both taxa are located dorsally within the body of the mandible and near the external dorsal margin.

Unlike the other taxa studied here, individual dorsal branches are visible in both mysticete mandibles. In all three taxa, these dorsal branches are extremely small in diameter. In the models shown in Figs 8–11, the branches appear discontinuous, but we consider this the result of a low sample resolution. In all three taxa the dorsal branches first appear anterior to the coronoid process just as the mandibular canal is deflecting towards the dorsal margin. In *Balaenoptera acutorostrata*, the dorsal branches are visible along the entire length of the mandible. In contrast, the dorsal branches of *Megaptera novaeangliae* are only visible at the proximal region (just anterior to the coronoid process) and in the distal one third of the mandible. While it is possible that this pattern is taxonomic, or even ontogenetic, we consider it most likely to be the result of the middle dorsal branches in *Megaptera novaeangliae* being too small to detect in the scan. Likewise, in the mandible of *Caperea marginata*, the dorsal branches are restricted to the proximal and distal ends. However, this mandible is broken in the middle such that the mandibular canal is exposed to the surface. Thus, it is likely that these dorsal branches were present throughout but are broken away with the rest of the morphology in this region. In the two rorqual mandibles, the proximal most dorsal branches are initially directed dorsally, but gradually angle anteriorly. Moving distally, each succeeding dorsal branch is increasingly angled anteriorly so that the distalmost dorsal branches have only the slightest dorsal deflection. In *Caperea marginata*, all of the observed dorsal branches extend directly anteriorly, or in some cases anteroventrally, with no dorsal deflection whatsoever. For all three, this pattern results in the dorsal branches exiting the dorsal margin of the mandible well anterior to their origination point within the mandibular canal. Each dorsal branch communicates directly with the dorsal margin of the mandible, resulting on a small foramen and associated sulcus on the dorsal surface. All together, these sulci make up the so called alveolar gutter” of the mysticete mandible.

## Discussion

### Internal morphology

Our results confirm that the foramina and sulci observed on the external surface of the mandibles of extant Mysticeti have patent internal connections to the mandibular canal, suggesting that they are functionally relevant. The mysticete mandibles in this study differ from those of other mammals examined herein in three key ways: they lack the dorsoventral expansion of the mandibular foramen characteristic of stem cetaceans and odontocetes; the dorsal branches of the mandibular canal are present as clearly ossified canals; and the mandibular canal is located dorsally within the body of the mandible and is close to the external dorsal margin. The latter two are particularly crucial to the issue of homology.

The condition observed in mysticetes, with the mandibular canal high in the body of the mandible, is reminiscent of that observed in edentulous human mandibles. The loss of adult dentition triggers trabecular bone remodeling and resorption in human mandibles [41–43]. It is possible that mysticetes recruit similar processes to modify their mandibles following the resorption of their embryonic lower tooth buds. Evidence of bone resorption is not surprising given that fetal mysticetes develop and resorb their dentition [7]. The postnatal mysticetes observed in this study show an alveolar gutter that is nearly closed (remaining open only at the dorsal foramen), and the dorsal margin in cross section is reminiscent of the latter stages shown by Ulm et al. [42]. This morphology, combined with the overall dorsal position of the mandibular canal, the complete lack of alveolar bone, and the known resorption of teeth *in utero*, all suggest extensive remodeling of the dorsal margin of the mandible in mysticete ontogeny. It is well documented that cranial bony elements are constantly remodeling in response to the stresses and strains of mastication [44, 45], and it is thus unsurprising that jaws freed from such stresses in an aquatic environment would remodel to an altogether different morphology. The lack of alveolar bone in particular is consistent with studies on humans and pigs demonstrating that, where teeth fail to develop, alveolar bone is absent and only basal bone is present [46, 47] Moreover, we observed this condition in all three mandibles studied here. Field et al. [48] reported a total length of 8 m for the humpback studied herein, which Huang et al. [49] considered as the approximate size class for weaning. This evidence suggests that bone resorption in mysticete mandibles occurs early in ontogeny, likely *in utero* when the tooth buds are resorbed.

### Evaluation of homology

The evidence for resorption calls into question the hypothesis that the dorsal foramina and associated sulci on mysticete mandibles are homologous to the alveolar branches of the mandibular canal. Our data confirm the presence of a continuous canal from the foramina to the mandibular canal, which is crucial for the viability of this hypothesis. However, this continuous canal is insufficient to establish homology to the alveolar branches alone; it merely confirms that the foramina allow for the passage of branches of the inferior alveolar artery and/or nerve. While the hypothesis of homology would seem more parsimonious (homology with a known structure as opposed to indicative of a unique structure), there are several reasons to doubt its viability.

Notably, this study failed to identify alveolar branches of the mandibular canal as ossified canals in either the terrestrial artiodactyls or the toothed cetaceans. Because the presence of these alveolar branches is well established [24, 25, 28], it is likely that they are not observed here because they are not contained within ossified canals as predicted by several authors [29, 30]. Moreover, the dorsal branches are strongly inclined anteriorly, so much so that they exit the mandible well anterior of their origination point at the mandibular canal. This pattern is those of a typical alveolar branch in toothed mammals, where the structures originate directly below the tooth root and projects dorsally into the pulp cavity. Altogether, the dorsal branches observed in mysticetes bear a distinct morphology than the alveolar branches of toothed taxa, calling their possible homology into question.

Moreover, homology between dorsal foramina and tooth alveoli is inconsistent both with existing mysticete tooth bud developmental data and the pattern of resorption observed in toothed taxa (including mysticetes *in utero*). Mysticete tooth buds pass through the bud, cap, and bell stages of development, without ever reaching the crown or maturation stage [7, 8, 50, 51]. These first three stages of tooth development occur within the alveolar process (inside trabecular bone); only during the final maturation stage does the developing tooth erupt [47]. Because mysticete tooth buds do not erupt in embryonic stages, there is no reason for the teeth, nor any associated vascular or nervous structure, to breach the dorsal surface of the mandible. Moreover, the position of the mandibular canal near the dorsal surface of the mandible indicates resorption of the mandibular bone is occurring in conjunction with tooth bud resorption. Therefore, the hypothesis of homology would suggest that resorption is extensive enough to degenerate the tooth buds and to nearly completely close the alveolar gutter, but ceases prior to eliminating the superficial foramina and sulci and filling in the internal ossified canals. To our knowledge, there is no other instance nor developmental model to explain a pause of the resorption process needed for the hypothesis of homology to be viable.

Lastly, such a pause of the resorption process would result in a morphology inconsistent with that observed for other edentulous mandibles [42, 43, 52]. The hypothesis of homology, with its required pause of bone resorption, would suggest either: 1) that branches of the inferior alveolar nerve and artery remain in the canals and travel through the mandible to no destination whatsoever (as there is no longer a pulp cavity or tooth), or; 2) that the soft tissue structures are resorbed without any associated remodeling of the bone, leaving empty canals. Neither of these implications is consistent with the morphology of edentulous mandibles following bone resorption [25, 43, 52].

Given this evidence, we consider the homology of the dorsal foramina in mysticete mandibles to the alveolar branches of other mammals tenuous at best. The dorsal branches of mysticete mandibular nerves are notably different in overall morphology compared to alveolar branches of toothed mammals, particularly in orientation, direction, and size. Moreover, the clear evidence of bone resorption and the lack of alveolar bone entirely suggest a complex remodeling process acting as a result of tooth bud resorption. If the dorsal branches are homologous with alveolar branches of toothed mammals than they are highly modified in overall morphology, path, and likely function. Thus, barring a detailed mechanical and developmental model to explain how the resorption of teeth interfaces with the observed morphology, we tentatively reject the hypothesis of homology. In particular, study of fetal specimens could theoretically bear on the issue. However, given the challenges associated with acquiring voucher specimens of mysticete fetuses, such a study would be difficult. Barring a thorough histological study of mysticete fetuses, the alveolar branches of mysticete tooth buds are unlikely to be observed, and their relationship to the dorsal branches of adult mysticetes difficult to track ontologically.

### Alternate explanations

By rejecting the hypothesis of homology, we suggest that the foramina observed on dorsal surface of the mandible represent a morphology associated with a distinct structure unique to crown (and possibly stem, edentulous) mysticetes (Fig 1). This hypothesis does not imply a pause in the resorption process, nor does it pose morphological variations such as empty passageways in the bone or nervous and vascular tissue with no destinations. Instead, we propose that novel branches of the inferior alveolar nerve and artery likely have a functional component under selection. While this hypothesis is more complex in suggesting a distinct structure, it is simpler in that it more consistently integrates with the established patterns associated with bone resorption in the edentulous mandible.

Notably, the suggestion of a novel sensory or vascular structure in the mysticete mandible is not without precedent. Pyenson et al. [13] reported the discovery of a sensory organ in the rorqual chin that facilitates and coordinates lunge feeding. They suggested that this sensory organ is innervated via the “relictual alveolar foramen” through expansion of the distalmost dorsal foramen. Though the sensory organ identified by Pyenson et al. [13] is known only in rorquals, it serves as a documented example of a novel soft tissue structure in mysticetes innervated by branches of the inferior alveolar nerve via the dorsal foramen. Accordingly, there is precedent for the type of evolution of a novel structure as suggested by our alternate hypothesis.

At the moment, our alternate hypothesis lacks a functional component and is not supported by phylogenetic comparisons. While the sensory organ identified in rorquals serves as a precedent for novel nervous structures, it has not been identified across all of crown Mysticeti that equally have the dorsal foramina, nor does it provide a functional explanation for all but the distalmost foramen. In their supplemental dataset, Pyenson et al. [9] noted that non-rorqual mysticetes possess vascular and nervous structures in the mandibular symphysis, yet lack a discrete cavity in which to organize them. It is possible that vascular and nerve branches from the distalmost alveolus permit low-level or incipient somatosensation for non-rorqual mysticete lineages, and that this condition was present at the node of crown Mysticeti prior to the evolution of subsequent innovations described for rorquals.

If there was selection for increased somatosensation on the dorsal margin of the lower jaw, it is possible that these branches provide sensory feedback during the filter feeding process. While the supporting tissue for baleen racks are innervated by the palate, individual baleen plates are not [53]. Optimal gape for precise placement of baleen relative to the lower jaw is likely essential for efficient filtration [54], and thus we suggest that one functional role of these dorsal branches may be to provide tactile sensory feedback regarding the alignment of the lower jaw relative to the baleen plates at the final stages of filtration.

Histological work would be particularly valuable in providing a functional explanation for these the dorsal branches. Such work should verify that the dorsal branches do contain soft tissue, and if so, verify if this tissue is vascular, nervous, or both. Additionally, we call for a more comprehensive comparative study documenting the variability in dorsal foramen morphology across both extant and extinct Mysticeti and suggest that the full variability of this character be placed in proper phylogenetic context to characterize how and when this morphology evolved. Strong emphasis should be placed on stem, edentulous mysticetes, because the presence of a stem mysticete lacking both teeth and dorsal foramina would strongly bolster the alternate hypothesis and imply novel branches unrelated to those associated with alveoli. Additionally, odontocetes specialized for suction feeding may serve as analogs for testing this hypothesis, as they too coordinate highly specialized oral movements that may require precise sensory feedback.

## Conclusions

We used computed tomography 3D models of the internal structures of the mandibles of fossil and living cetaceans, along with several terrestrial sister taxa, to assess the morphological consequences of tooth loss in mysticete cetaceans. We demonstrate that the foramina visible on the dorsomedial surface of mysticete mandibles bear internal canals connecting them to the mandibular canal. In doing so, we provide an evidence-based approach to test the homology of these dorsal canals to the alveolar branches of terrestrial mammals.

We identify a pattern of resorption within the mandibles of mysticete cetaceans reminiscent of that observed in terrestrial mammals following the loss of the adult dentition. This resorption process results in the mandibular canal positioned high in the body of the mandible unlike the condition seen in the mandibles of other terrestrial mammals. The presence of clear, ossified canals, despite evidence for bone resorption, is inconsistent with established models for bone resorption following the loss of the adult dentition in toothed mammals.

If the dorsal branches of mysticete mandibles are homologous to alveolar branches of other mammals, we argue that this connection requires a cessation of the resorption process in mysticete ontogeny. Accordingly, this would suggest that the dorsal canals contain soft tissue without a destination and/or function, or that the soft tissue is resorbed but the canals are not, leaving empty canals in the bone. Neither of these possibilities is likely, given established mammalian responses to bone remodeling. Alternatively, we argue that the observed dorsal branches represent distinct branches, unrelated to the alveolar branches supplying the pulp cavity of teeth; this hypothesis has clear precedent in the mandibular sensory organ documented by Pyenson et al. [13].

We call for more rigorous examination of the soft tissue of the mysticete mandible to identify the function of these canals (if any exists) as a means of further testing these and other functional hypotheses. Soft tissue dissections or imaging techniques should be used to identify whether soft tissue structures are still present in the alveolar branches of mysticetes and, if so, whether they are nervous, vascular, or both. A lack of nervous or vascular tissue in the canals would imply that the canals are truly vestigial, and would suggest an unusual halting of the resorption process. In contrast, the presence of nervous or vascular tissue in the dorsal branches would hint at a yet unknown function. It is possible that sensation to the soft tissue of the mandible bears some role in mysticete feeding. Future studies should aim to track and document the morphology and function of any potential soft tissue housed within the alveolar branches of the mandibular canal.
